# Statin Use Is Associated With a Decline in Muscle Function and Mass Over Time, Irrespective of Statin Pharmacogenomic Score

**DOI:** 10.1002/jcsm.70132

**Published:** 2025-11-20

**Authors:** Mélissa Gentreau, Mahitab Sakr, Salahuddin Mohammad, Ahmed M. Alsehli, Olga E. Titova, Gull Rukh, Helgi B. Schiöth

**Affiliations:** ^1^ Functional Pharmacology and Neuroscience, Department of Surgical Sciences Uppsala University Uppsala Sweden; ^2^ Department of Physiology, Faculty of Medicine King Abdulaziz University Jeddah Kingdom of Saudi Arabia; ^3^ Medical Epidemiology, Department of Surgical Sciences Uppsala University Uppsala Sweden

**Keywords:** aging, hydroxymethylglutaryl‐CoA reductase inhibitors, muscle mass, muscle strength, polygenic risk score, sarcopenia

## Abstract

**Background:**

Statins are cholesterol‐lowering drugs widely prescribed for preventing cardiovascular diseases. They may cause adverse effects on skeletal muscle, but it remains unclear whether they affect muscle function and mass. We aimed to evaluate the association between statin use and muscle function and mass, and whether the pharmacogenomic score (PGS) of statin response modifies these associations.

**Methods:**

We included 297 977 participants from the UK Biobank. Grip strength was measured using a Jamar J00105 hydraulic hand dynamometer, and the appendicular lean mass (ALM) was estimated using bioelectrical impedance analysis. We performed linear regression to evaluate the cross‐sectional and longitudinal associations between statin use and (changes in) grip strength or ALM, adjusting for demographic, lifestyle and health factors. We tested the interaction with the PGS and stratified the analysis by PGS tertile.

**Results:**

Participants averaged 56.4 (± 8) years, and 46% were male. Statin use was associated with lower baseline grip strength (β = −0.68 kg [−0.89, −0.48]) and ALM (β = −0.19 kg [−0.22, −0.16]). Among 35 557 participants with follow‐up data (10 ± 5 years), continuous statin use was associated with an accelerated decline in grip strength (β = −0.32 kg/year [−0.49, −0.14]) and ALM (β = −0.06 kg/year [−0.08, −0.03]) compared with never users. The PGS showed a potential modifying effect at baseline (*p* = 0.058 for grip strength and *p* = 0.068 for ALM) but did not significantly influence the rate of decline over time.

**Conclusions:**

Continuous statin use is associated with a decline in muscle function and mass over time (25% decline in grip strength and 73% decline in ALM compared to never‐users), irrespective of genetic susceptibility to statin response. This study emphasizes the importance of monitoring musculoskeletal health in statin users and supports further research into the potential role of a healthy diet and regular physical activity in preserving muscle function, which may also reinforce the cardiovascular benefits of statin therapy.

## 1 Introduction

Statins are among the most effective and widely prescribed lipid‐lowering agents, playing a pivotal role in cardiovascular disease prevention. By inhibiting 3‐hydroxy‐3‐methylglutaryl coenzyme A (HMG‐CoA) reductase, the rate‐limiting enzyme in cholesterol biosynthesis, statins reduce hepatic cholesterol levels, leading to upregulation of low‐density lipoprotein (LDL) receptors and enhanced clearance of circulating LDL cholesterol [1]. Beyond their cholesterol‐lowering properties, statins exert pleiotropic effects, including anti‐inflammatory, antioxidant and endothelial‐stabilizing benefits, contributing to plaque stabilization and reduced cardiovascular events [2].

Despite their efficacy, statin therapy is associated with adverse effects on skeletal muscle, ranging from mild myalgia to severe myopathy, often accompanied by significantly elevated creatine kinase levels [3]. However, whether statin use impacts muscle function and/or mass remains unclear, with conflicting findings in the literature. Some cross‐sectional studies reported a negative association between statin use and grip strength, suggesting that statins may contribute to muscle weakness in older adults [4], while others found no association [5, 6, 7]. Longitudinal studies have similarly provided mixed results. For example, some studies suggested that statin use was associated with a decline in leg strength and muscle quality [8] and may impair muscle strength recovery after events such as stroke [9]. In contrast, another study reported that statin users performed slightly better than non‐users on timed chair stands, with a significant association observed among participants under 80, who had fewer co‐morbidities and medications [10]. Two studies found no significant changes in grip strength over time [11, 12]. These inconsistencies suggest that the relationship between statins and musculoskeletal health may depend on individual characteristics such as age, co‐morbidities and medication burden.

Moreover, genetic factors may contribute to inter‐individual variability in therapeutic response and risk of adverse effects. Advances in pharmacogenomics have highlighted the role of genetic variation in modulating statin efficacy and the risk of adverse effects, including muscle symptoms [13] and hepatotoxicity, which is manifested by transient liver enzyme elevations, although severe damage is rare [14]. Single‐nucleotide polymorphisms (SNPs) in genes involved in statin metabolism (e.g., *CYP3A4* and *CYP2C9*), transport (e.g., *SLCO1B1* and *ABCG2*) and pharmacodynamics (e.g., *APOE* and *PCSK9*) have been implicated in statin‐associated muscle symptoms.^15,S1^ However, no comprehensive approach has been developed to quantify individual genetic susceptibility to statin response.

To address this, we constructed a pharmacogenomic score (PGS) aggregating genetic variants associated with statin response. This polygenic approach incorporates SNPs strongly associated with statin metabolism, LDL‐lowering efficacy, myopathy risk and hepatic enzyme elevations identified in genome‐wide association studies (GWAS). By integrating genetic predisposition, this score provides an individualized measure of susceptibility to statin response. Using data from the UK Biobank, our study aimed to determine whether genetic predisposition influences the association between statin use and muscle‐related outcomes. Specifically, we evaluated the association of statin use with muscle function and mass, as measured by grip strength and appendicular lean mass (ALM), in cross‐sectional and longitudinal analyses (changes over time), and we assessed whether genetic predisposition, as captured by the PGS, modified these associations.

## 2 Methods

### 2.1 Study Sample

The UK Biobank is a prospective cohort of around 500 000 middle‐aged and older participants across the United Kingdom. Baseline data collection between 2006 and 2010 included sociodemographic and lifestyle information, medical history, medication use, physical measurements and biological samples.^S2^ In 2014, a large follow‐up visit started and is still ongoing. Data released in January 2024 included more than 75 000 participants who completed the second visit. The UK Biobank project received ethical approval from the North West Multi‐center Research Ethics Committee. Additionally, the Regional Ethics Committee of Uppsala (Sweden) approved the study for data use. The current study excluded participants either lacking medication information at baseline (*n* = 863) or using both statins and other lipid‐lowering medications (*n* = 5357). Besides, we excluded participants having prevalent organic mental disorders (*n* = 15 067) and neuromuscular‐related diseases (*n* = 37 880) defined by the International Classification of Diseases‐10^th^ revision. Finally, since we made use of the genetic data, we excluded participants with missing genetic data (*n* = 12 564), failed quality control of the genotyped data (*n* = 70 439), non‐European ancestry (*n* = 62 081), and for whom we were unable to calculate the PGS (*n* = 15). From the 49 837 participants completing the second visit, the longitudinal study excluded participants lacking medication information (*n* = 6421) or who used both statins and other lipid‐lowering medications at the second visit (*n* = 191). Finally, participants who stopped using statins (*n* = 561) or started using statins at the second visit (*n* = 7107) were excluded. The final study sample included 297 977 participants for the cross‐sectional analysis and 35 557 for the longitudinal analysis (Figure S1).

### 2.2 Statin Use

Statin use was reported at baseline and the second follow‐up visit using a touchscreen questionnaire, confirmed during a verbal interview, and recorded as medication codes. Statin medication codes were extracted and transformed into a single binary variable (statin use: yes/no). A similar process was used to extract data on non‐statin cholesterol‐lowering medications.^S3^

### 2.3 Grip Strength Measurement

Grip strength is a surrogate marker of muscle function and is recommended for assessing general muscular strength by the European Working Group on Sarcopenia in Older People (EWGSOP2).^S4^ Right and left grip strength were measured at baseline and follow‐up visits using a Jamar J00105 hydraulic hand dynamometer. Participants were instructed to perform the test seated with the arm at a 90° angle, following standard procedures. Total grip strength (kg) was calculated as the sum of the measurements from both hands.

### 2.4 Appendicular Lean Mass Calculation

Appendicular lean mass (ALM) reflects the lean tissue mass of the limbs, which are major sites of skeletal muscle storage. It was used as a proxy for muscle mass and calculated as previously described [15]. Briefly, ALM was determined at baseline and follow‐up visits by summing the fat‐free mass of the arms and legs, estimated using the bioelectrical impedance analysis (BIA) method. BIA fat‐free mass showed a strong correlation (r = 0.96) with body composition measured by dual‐energy X‐ray absorptiometry (DXA) in a subset of participants.^S5^

### 2.5 Pharmacogenomic Score Computation

The PGS was computed as a weighted sum of risk alleles across genetic variants using GWAS.^S6^ UK Biobank genetic data included genotyped, imputed and quality‐controlled data for over 90 million genetic variants with genotyping performed using the UK BiLEVE and UK Biobank Axiom arrays [16]. We extracted summary statistics from the GWAS Catalogue (accessed on April 30, 2024) from European ancestry studies to list SNPs associated with statin response traits (Table S1) and to build a genetic score of statin response. There was no participant overlap between the GWAS selected and the UK Biobank. We checked that the allele frequencies of the SNPs in the UK Biobank were similar to the GWAS. We excluded SNPs with a minor allele frequency (MAF) < 1%, those that did not verify Hardy–Weinberg equilibrium (*p* < 1 × 10^−6^), and dependent SNPs according to linkage disequilibrium (r^2^ > 0.8)

ThePGSwas calculatedas:

$$\sum_{i=1}^{N}\beta_{i}\times G_{i}$$

where N is the number of SNPs, $\beta_{i}$ the effect size from GWAS summary statistics, and $G_{i}$ the genotype dosage (0, 1 or 2).

SNP extraction and PGS calculation were performed using PLINK software (version 1.9).

### 2.6 Covariates

At the baseline assessment, general information was collected on the touchscreen questionnaire. Education level was recoded from the education qualifications data as having obtained a college or university degree. The Townsend deprivation index (TDI) was used as a proxy of socioeconomic status.

Smoking and drinking status were defined as never, previous or current. Body mass index (BMI) was calculated from measured weight/height^2^ (kg/m^2^). The total Metabolic Equivalent Task score (MET, in min/week) was calculated according to International Physical Activity Questionnaire guidelines, including all activities: mild (walking), moderate (running) and vigorous [17]. The MET score was then recoded to inactive (< 600 min/week), active (600 to < 1200 min/week), and highly active (≥ 1200 min/week) [18]. Diet quality was based on a self‐reported questionnaire where the frequency of intake was reported for 14 common food groups. A healthy diet was defined as a high consumption of fruits (fresh and dried fruits), vegetables (raw and cooked vegetables), and oily fish and a low consumption of processed and red meat (processed meat, beef, lamb/mutton, pork) and cheese, as recommended by the American Heart Association.^S7^ We used the sex‐specific median as cut‐offs (Table S2). The total diet quality score ranged from 0 (*lowest quality*) to 10 (*highest quality*).

Medical history information, extracted from the verbal interview, included depression, hypertension, diabetes, coronary heart disease (angina and/or heart attack), liver failure/cirrhosis, chronic obstructive airway disease/COPD and renal/kidney failure (no/yes).

### 2.7 Statistical Analysis

All statistical analyses were conducted using R version 4.4.1 (R Foundation for Statistical Computing, Vienna, Austria).

#### 2.7.1 Association Between Statin Use and Muscle Outcomes at Baseline

We performed linear regression models to evaluate the cross‐sectional association between statin use and grip strength or ALM assessed at baseline. Models were adjusted for assessment center, age, sex, education, TDI, smoking and drinking status, BMI, MET score, diet quality score, history of depression, hypertension, diabetes, coronary heart disease, liver failure/cirrhosis, chronic obstructive airway diseases/COPD and renal/kidney failure. Covariates were selected based on prior literature as potential common causes of both statin use and muscle function/mass variation [19, 20].

##### 2.7.1.1 Multiple Imputation of Missing Values

Missing values among covariates varied between 0% and 21.6%. To address this, we applied multiple imputation via the chained equations using the ‘mice’ R package with the random forest method [21]. We generated eight imputed datasets, analysed them separately and pooled the results using Rubin's rules.

##### 2.7.1.2 Propensity Score Matching

To test the robustness of our cross‐sectional analysis, we performed a propensity score matching before and after multiple imputations of missing values. We estimated the propensity score for statin use using a logistic regression model with the same covariates as in the above analysis. Statin users were matched one‐to‐one with non‐users using the nearest‐neighbour algorithm without replacement with the ‘MatchIt’ R package [22]. Individuals without an appropriate match were excluded. The standardized mean differences were computed before and after matching to check balance. A threshold below 0.1 was considered adequate.^S8^ Finally, the linear regression was performed on the matched sample, further adjusting for covariates to minimize residual confounding.

#### 2.7.2 Association Between Continuous Statin use and Changes in Muscle Outcomes Over Time

We evaluated whether continuous statin use (i.e., reported at both baseline and follow‐up) was associated with changes in grip strength and ALM over time. First, changes in grip strength or ALM were calculated as the difference between follow‐up and baseline measures. Time was calculated as the time difference (years) between the two assessments. Then, we built linear regression models with changes in grip strength or ALM as the outcome, and statin use, time and the interaction term between statin and time as exposure variables. Models were adjusted for the same covariates as in the cross‐sectional analysis. The β estimate for statin use represents the difference in baseline changes in grip strength or ALM between continuous statin users and never users. The β estimate for time represents the annual rate of change in grip strength or ALM. Finally, the β estimate for the interaction term (statin use*time) indicates whether the annual rate of change in grip strength or ALM (expressed as kg/year) differs between continuous statin users and never users. In sensitivity analysis, these analyses were further replicated after the multiple imputation of missing values.

#### 2.7.3 Sensitivity Analyses

To assess potential confounding by glycaemic status and blood pressure medications, we performed sensitivity analyses that additionally adjusted the cross‐sectional and longitudinal models for [1] glycaemic status categorized using HbA1c (normoglycaemia: < 42 mmol/mol; prediabetes: 42–47 mmol/mol; diabetes: ≥ 48 mmol/mol or diabetes medication) and [2] hypertension or medication for blood pressure.

Additionally, we performed sensitivity analyses using ALM derived from DXA to confirm the BIA‐based results. DXA measurements were available at the second (*n* = 40 278) and third visits (*n* = 4016). For these analyses, the second visit was treated as the baseline. Cross‐sectional models included all covariates as in the main analysis, and longitudinal models examined changes between the second and third visits.

#### 2.7.4 Modification Effect by the Pharmacogenomic Score

For both cross‐sectional and longitudinal analyses, we assessed whether genetic predisposition to statin response, as captured by the PGS, modified the association between statin use and muscle outcomes. First, we validated the PGS by assessing its association with lipid‐related blood biomarkers using linear regression adjusted for SNP chip (i.e., UK BiLEVE or UK Biobank Axiom arrays), the 10 genetic principal components, assessment center, age and sex. Then, we tested the statistical interaction between statin use and the PGS and stratified the analysis by PGS tertile (Low, Medium, High). Models were adjusted for the SNP chip, the 10 genetic principal components, and the same covariates used in the previous analyses. To investigate whether findings were driven by a specific SNP or reflected a cumulative effect, a leave‐one‐out analysis was conducted by sequentially omitting one SNP from the PGS and recalculating the interaction between statin use and PGS.

#### 2.7.5 Modification Effect by Lifestyle Factors

Finally, we conducted an exploratory analysis to assess whether lifestyle factors, such as diet quality and physical activity, modified the association between statin use and changes in muscle outcomes. Diet quality and MET scores were used to categorize participants into high vs. low diet quality/physical activity groups throughout the follow‐up. Interaction terms between statin use, time and lifestyle categories were tested.

## 3 Results

A total of 297 977 were included in the study sample at baseline. Statin users were older (61.4 vs. 55.7 years), more often male (62.8% vs. 43.4%), and had a higher BMI (29.2 vs. 26.9) compared to non‐users. They were less likely to hold a college or university degree and more likely to present co‐morbidities and to take medications for blood pressure or diabetes (Table 1).

**TABLE 1:** Characteristics of the study sample at baseline.

Characteristics	All sample	Statin non‐users	Statin users
Mean (SD) or *n* (%)	*n* = 297 977	*n* = 258 598	*n* = 39 379
Age	56.4 (8)	55.7 (8)	61.4 (6)
Sex (men)	137 108 (46)	112 359 (43.4)	24 749 (62.8)
College or university degree	96 988 (32.5)	87 544 (33.9)	9444 (24)
TDI^a^	−1.6 (2.9)	−1.7 (2.9)	−1.3 (3.1)
BMI (kg/m^2^)	27.2 (4.7)	26.9 (4.5)	29.2 (4.9)
MET score			
< 600 min/week	41 298 (13.9)	35 109 (13.6)	6189 (15.7)
600 to < 1200 min/week	41 148 (13.8)	35 725 (13.8)	5423 (13.8)
≥ 1200 min/week	151 271 (50.8)	133 170 (51.5)	18 101 (46)
Smoking status			
Never	165 193 (55.4)	147 808 (57.2)	17 385 (44.1)
Previous	102 564 (34.4)	84 973 (32.9)	17 591 (44.7)
Current	29 281 (9.8)	25 072 (9.7)	4209 (10.7)
Alcohol status			
Never	8621 (2.9)	7154 (2.8)	1467 (3.7)
Previous	9241 (3.1)	7601 (2.9)	1640 (4.2)
Current	279 889 (93.9)	243 650 (94.2)	36 239 (92)
Diet quality	5.5 (1.7)	5.5 (1.7)	5.4 (1.7)
Hypertension	72 390 (24.3)	48 401 (18.7)	23 989 (60.9)
Diabetes	9365 (3.1)	2835 (1.1)	6530 (16.6)
Coronary heart diseases	10 774 (3.6)	2269 (0.9)	8505 (21.6)
Chronic obstructive airway diseases/COPD	906 (0.3)	676 (0.3)	230 (0.6)
Liver failure/cirrhosis	169 (0.1)	146 (0.1)	23 (0.1)
Renal/kidney failure	102 (0)	59 (0)	43 (0.1)
Depression	15 974 (5.4)	13 665 (5.3)	2309 (5.9)
Medications for blood pressure	56 111 (18.8)	31 378 (12.1)	24 733 (62.8)
Medications for diabetes	2143 (0.7)	725 (0.3)	1418 (3.6)
HbA1c			
< 42 mmol/mol	265 581 (89.1)	236 860 (91.6)	28 721 (72.9)
42–47 mmol/mol	10 630 (3.6)	6599 (2.6)	4031 (10.2)
≥ 48 mmol/mol	7814 (2.6)	3004 (1.2)	4810 (12.2)

^a^ Median (interquartile range).

### 3.1 Statin Users Have Lower Grip Strength and Appendicular Lean Mass Than Non‐Users

We used linear regression models to examine the cross‐sectional association between statin use and grip strength or ALM (Table 2). In fully adjusted models, statin use was associated with lower grip strength (β = −0.683 kg [−0.888, −0.478]; *p* = 6e‐11) and lower ALM (β = −0.188 kg [−0.220, −0.155]; *p* < 1e‐16).

**TABLE 2:** Cross‐sectional association of statin use with baseline grip strength and appendicular lean mass.

Outcome	β	95% confidence interval		*p*
		Lower	Upper	
Grip strength (kg)				
Unadjusted	1.383	1.149	1.617	< 1e‐16
Adjusted	−0.683	−0.888	−0.478	6e‐11
Appendicular lean mass (kg)				
Unadjusted	2.029	1.969	2.089	< 1e‐16
Adjusted	−0.188	−0.22	−0.155	< 1e‐16

*Note:* Linear model adjusted for assessment center, age, sex, education, TDI, smoking and drinking status, BMI, MET score, diet quality score, history of depression, hypertension, diabetes, coronary heart disease, liver failure/cirrhosis, chronic obstructive airway diseases/COPD, renal/kidney failure.

To assess the robustness of our findings, we applied propensity‐score matching prior to regression analyses. This matching created balanced groups of statin users and non‐users (*n* = 31 328 per group), with standardized mean differences for all covariates below 10% except for CHD (~15%), suggesting some residual confounding (Figures S2 and S3). After imputation of missing values, the matched sample increased to 39 148 per group. Across all approaches, the inverse association between statin use and both grip strength and ALM remained consistent (Table S3).

### 3.2 Continuous Statin Use Is Associated With a Decline in Grip Strength and Appendicular Lean Mass Over Time

**FIGURE 1:**
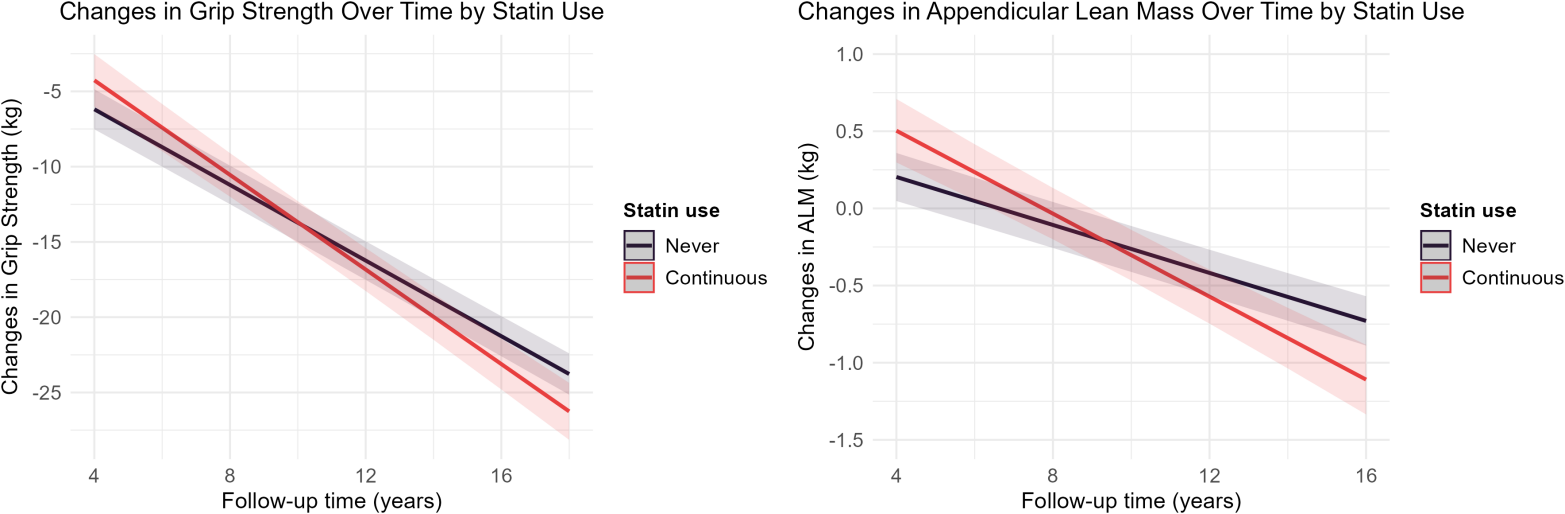
Changes in grip strength and appendicular lean mass over time by statin use. Predicted changes in grip strength (left) and appendicular lean mass (right) with a 95% confidence interval were obtained from the linear model adjusted for assessment center, age, sex, education, TDI, smoking and drinking status, BMI, MET score, diet quality score, history of depression, hypertension, diabetes, coronary heart disease, liver failure/cirrhosis, chronic obstructive airway diseases/COPD, renal/kidney failure.

We evaluated whether continuous statin use was associated with changes in grip strength and ALM between baseline and follow‐up using linear regression with an interaction term between statin use and time. Continuous statin use was significantly associated with an accelerated decline in grip strength (β = −0.315 kg/year [−0.485, −0.144]; *p* = 3e‐04) and ALM (β = −0.057 kg/year [−0.081, −0.033]; *p* = 4e‐06), despite higher baseline values (Table 3). Predicted changes in grip strength and ALM over time identified a crossover point around 10 years of follow‐up. At this point, the initial advantage observed in statin users diminishes over time, and their grip strength and ALM fall behind those of never‐users (Figure 1). Results were unchanged after multiple imputation (Table S4).

**TABLE 3:** Association between continuous statin use and change in grip strength and appendicular lean mass over 10‐year follow‐up.

Outcome	β	95% confidence interval		*p*
		Lower	Upper	
Changes in grip strength (kg)				
Statin use	3.183	1.33	5.036	8e‐04
Time	−1.256	−1.319	−1.192	< 1e‐16
Statin use*Time	−0.315	−0.485	−0.144	3e‐04
Changes in appendicular lean mass (kg)				
Statin use	0.527	0.29	0.763	1e‐05
Time	−0.078	−0.086	−0.069	< 1e‐16
Statin use*Time	−0.057	−0.081	−0.033	4e‐06

*Note:* Statin use is defined as continuous use between baseline and follow‐up versus never (reference). Linear model adjusted for assessment center, age, sex, education, TDI, smoking and drinking status, BMI, MET score, diet quality score, history of depression, hypertension, diabetes, coronary heart disease, liver failure/cirrhosis, chronic obstructive airway diseases/COPD, renal/kidney failure.

### 3.3 Sensitivity Analysis

Additional adjustments were made for glycaemic status and blood pressure medications, and the results were similar to those of the main analyses (Table S5). Sensitivity analysis using DXA‐derived ALM also confirmed the main BIA‐based findings (Table S6).

### 3.4 The Pharmacogenomic Score Modifies the Cross‐Sectional Association but Not the Longitudinal Association Between Statin Use and Muscle Outcomes

**FIGURE 2:**
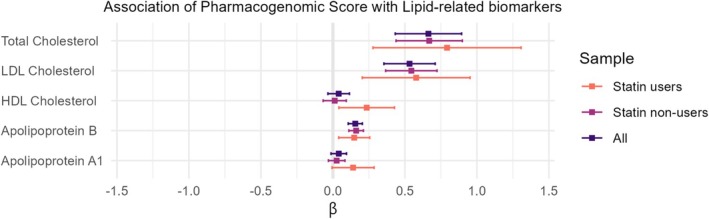
Association between the pharmacogenomic score of statin response and lipid‐related biomarkers in the blood. The β estimate and 95% confidence interval were obtained from the linear model adjusted for SNP chip, the 10 genetic principal components, the assessment center, age, and sex.

The PGS included SNPs related to statin response, with 12 (26%) associated with LDL‐cholesterol change and 11 (24%) with simvastatin‐induced myopathy; the three most influential SNPs (rs4256319, rs140854723 and rs184787123) had the largest absolute effect sizes despite low MAF (Figure S4). We first tested whether the PGS was associated with lipid particle concentration in the blood, due to the strong contribution of SNPs associated with LDL cholesterol change. Higher PGS was significantly associated with higher concentrations of total cholesterol, LDL cholesterol and apolipoprotein B (Figure 2).

**FIGURE 3:**
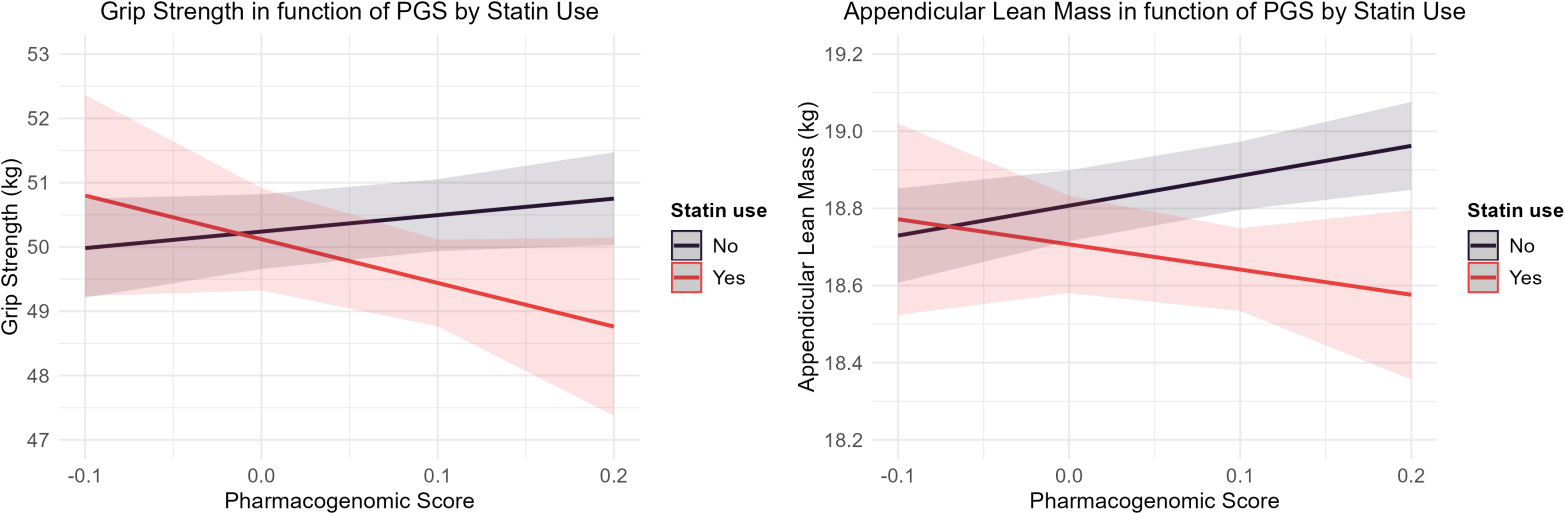
Grip strength and appendicular lean mass in function of pharmacogenomic score (PGS) by statin use. Predicted values of grip strength and appendicular lean mass with a 95% confidence interval were obtained from the linear model adjusted for SNP chip, the 10 genetic principal components, assessment center, age, sex, education, Townsend deprivation index, smoking and drinking status, BMI, MET score, diet quality score, history of depression, hypertension, diabetes, coronary heart disease, liver failure/cirrhosis, chronic obstructive airway diseases/COPD, renal/kidney failure.

**FIGURE 4:**
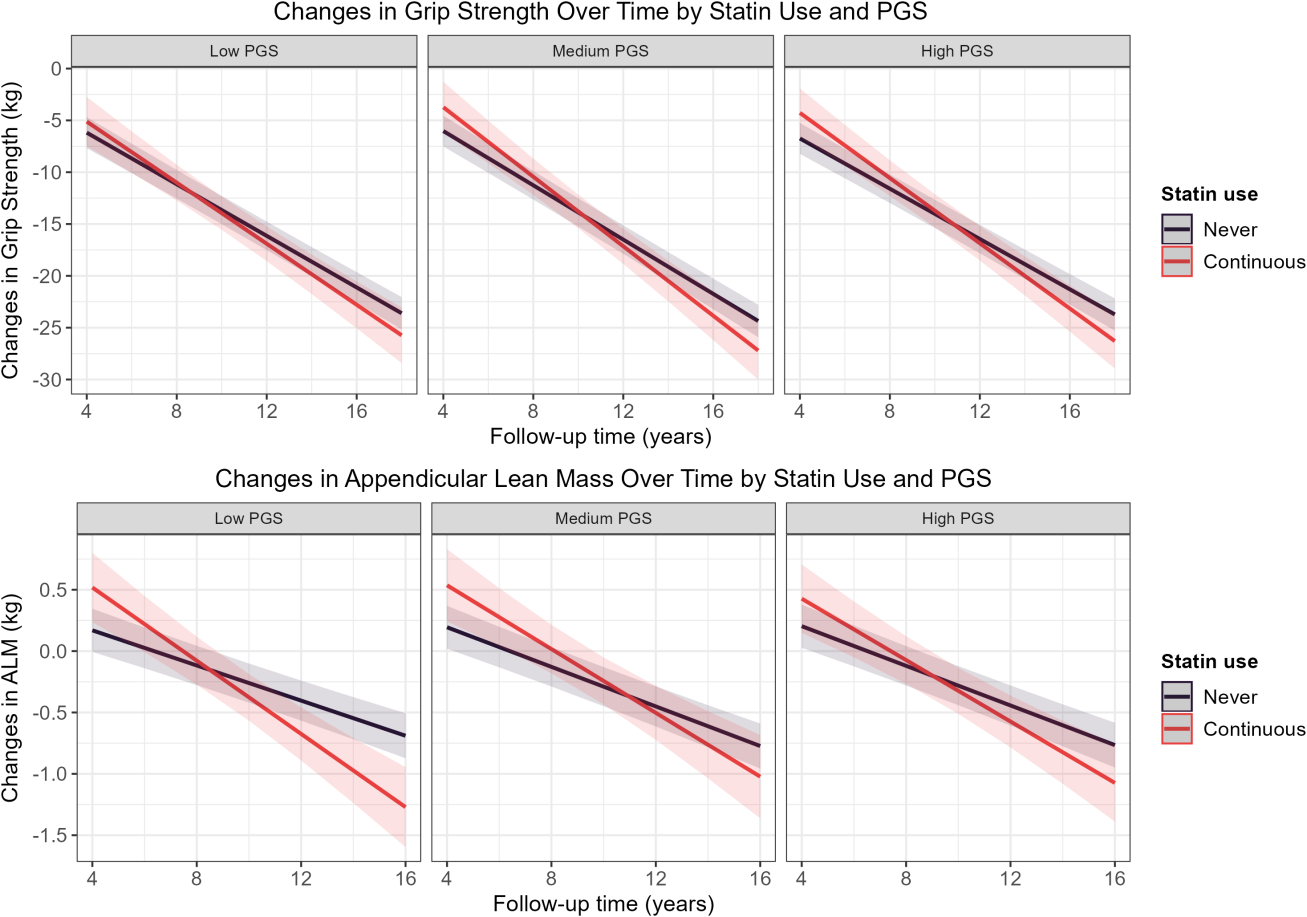
Changes in grip strength and appendicular lean mass over time by pharmacogenomic score (PGS) tertile and statin use. Predicted changes in grip strength and appendicular lean mass with a 95% confidence interval were obtained from the linear model adjusted for SNP chip, 10 genetic principal components, assessment center, age, sex, education, Townsend deprivation index, smoking and drinking status, BMI, MET score, diet quality score, history of depression, hypertension, diabetes, coronary heart disease, liver failure/cirrhosis, chronic obstructive airway diseases/COPD, renal/kidney failure.

Then, we tested the interaction between PGS and statin use on muscle outcomes. In cross‐sectional analyses, this interaction showed a trend toward significance for grip strength and ALM (*p* = 0.058 and *p* = 0.068, respectively), suggesting a possible modifying effect of PGS (Table S7). Predicted values and stratified analyses by PGS tertile indicated that statin users with the highest PGS had the lowest grip strength and ALM (Figure 3, Table 4). A leave‐one‐out analysis highlighted rs17815112 as an important contributor, particularly for ALM (Table S8). In longitudinal analyses, no significant interaction was observed for changes in grip strength or ALM, with predicted trajectories and stratified analyses showing consistent declines across PGS strata (Figure 4, Table S9).

**TABLE 4:** Cross‐sectional association of statin use with grip strength and appendicular lean mass stratified by the pharmacogenomic score tertile.

Outcome	β	95% Confidence Interval		*p*
		Lower	Upper	
Grip Strength (kg)				
Low PGS	−0.410	−0.764	−0.0558	0.023
Medium PGS	−0.806	−1.162	−0.451	9e‐06
High PGS	−0.859	−1.213	−0.506	2e‐06
Appendicular lean mass (kg)				
Low PGS	−0.165	−0.221	−0.109	9e‐09
Medium PGS	−0.168	−0.224	−0.111	5e‐09
High PGS	−0.228	−0.285	−0.172	2e‐15

*Note:* Linear model adjusted for SNP chip, the 10 genetic principal components, assessment center, age, sex, education, TDI, smoking and drinking status, BMI, MET score, diet quality score, history of depression, hypertension, diabetes, coronary heart disease, liver failure/cirrhosis, chronic obstructive airway diseases/COPD, renal/kidney failure.

### 3.5 Lifestyle Factors Might Modify the Association Between Statin Use and Changes in Muscle Function

Exploratory interaction analyses suggested that diet quality and physical activity might modify the association between continuous statin use and grip strength decline. Specifically, the predicted values indicated that continuous statin users with high diet quality (Figure S5, top) or high physical activity (Figure S6, top) had a slower decline in grip strength over time. However, no such observations were made for ALM, with no change for diet quality (Figure S5, bottom) and a faster decline with high physical activity (Figure S6, bottom).

## 4 Discussion

In this UK Biobank study, we primarily investigated whether statin use is associated with muscle function and mass, and whether statin PGS modifies these associations. Our results indicated that statin users had lower baseline grip strength and ALM than non‐users after adjustment for confounders and sensitivity analyses. Longitudinal analysis revealed that continuous statin use was associated with a faster decline in both grip strength and ALM, such that after approximately 10 years of follow‐up, statin users displayed lower trajectories of muscle function and mass than never users. Regarding genetic susceptibility to statin response, we observed a potential modifying effect of the PGS on the cross‐sectional association between statin use and muscle outcomes, with statin users with a high PGS displaying the lowest grip strength and ALM. However, this genetic susceptibility did not significantly alter the rate of decline in grip strength and ALM over time. Overall, this study shows that statin use is associated with an accelerated decline in muscle function and mass over time, irrespective of statin PGS.

Our finding that statin use is associated with lower baseline grip strength contrasts with most previous cross‐sectional studies, which reported no association [5, 6, 7], except for one that identified lower grip strength in statin users [4]. However, this study relied on a simple comparison between statin users (*n* = 77) and non‐users (*n* = 75), without adjustment for confounders [4], which could introduce bias and increase the likelihood of a chance finding. Other studies may have failed to adjust for confounders such as BMI [7] or co‐morbidities [5]. Some may have lacked statistical power [6] and had older participants [6, 7]. In contrast, our study benefits from a large sample and a sensitivity analysis matching statin users and non‐users. Moreover, we demonstrated that continuous statin use is associated with an accelerated decline in muscle function and mass over time. This association represents an additional 25% decline in grip strength and a 73% decline in ALM among continuous statin users compared to never‐users. These results partly align with findings from two longitudinal studies. In the Tasmanian Older Adult Cohort Study, continuous statin use was associated with a decline in leg strength and muscle quality, but no change in ALM [8]. Similarly, statin users recovering from a stroke who had sarcopenia displayed lower grip strength upon discharge, despite unchanged muscle mass [9]. However, two other studies found no significant association between statin use and grip strength changes over time and did not assess muscle mass [11, 12]. These discrepancies may stem from differences in follow‐up.

Interestingly, our longitudinal analysis showed that continuous statin users began their muscle strength and mass trajectories at a higher baseline level than non‐users. Several factors may help explain this. First, a higher proportion of statin users in the longitudinal sample were men (70%), and men generally exhibit greater grip strength and muscle mass than women [23] This imbalance may reflect disparities in statin prescription, with women less likely to receive statins due to the underestimation of their cardiovascular risk and their underrepresentation in clinical trials [24]. Second, selection bias may also contribute, as the longitudinal sample included slightly younger participants with fewer co‐morbidities (Table S10), indicating that statin users in the follow‐up sample may be a healthier, more resilient subgroup. This ‘healthy survivor’ effect could artificially inflate baseline grip strength and ALM estimates in longitudinal models, despite the observed accelerated decline over time.

Several biological mechanisms could underlie the associations we observed. Statins inhibit HMG‐CoA reductase, reducing not only cholesterol synthesis but also key intermediates of the mevalonate pathway, such as isoprenoids and coenzyme Q10 [3]. The depletion of these intermediates has been linked to impaired protein prenylation, mitochondrial dysfunction and inhibition of protein synthesis, all of which may contribute to muscle atrophy [25]. Additional mechanisms include increased apoptosis, disruption of calcium homeostasis and general myotoxicity reflected by elevated creatine kinase levels [3]. Evidence suggests preferential vulnerability of type II (fast‐twitch) fibres [26], which are glycolytic and primarily contribute to muscle mass [27], thereby contributing to the pronounced ALM decline we observed. In contrast, type I fibres, more oxidative and fatigue‐resistant [27], may partly preserve muscle endurance, consistent with attenuated grip strength loss in active statin users [26]. Beyond direct myotoxicity, statins may aggravate insulin resistance and diabetes risk [28], while severe muscle injury such as rhabdomyolysis can trigger systemic phosphate toxicity, a mechanism implicated in muscle loss, accelerated aging and broader pathological outcomes [29]. These pathways suggest that statin‐related muscle decline is not an isolated effect on muscle fibres, but part of a wider spectrum of metabolic disturbances.

By constructing the PGS, our study provides a comprehensive assessment of individual susceptibility to statin‐related outcomes, assessing the cumulative effect of genetic variants on the association between statin use and muscle outcomes. Although the PGS appeared to modify the baseline association between statin and muscle outcomes, it did not significantly modify the trajectory of muscle decline over time. Notably, rs4256319, rs140854723 and rs184787123, which contributed most to the PGS, did not change the interaction estimate, despite their known association with statin‐associated myopathy [30]. Likewise, rs4149056 (c.521 T > C, p.Val174Ala) in *SLCO1B1*, which encodes for the hepatic transporter OATP1B1 for statin uptake, did not modify the long‐term trajectory of muscle function and mass, despite its established link with statin‐associated muscle symptoms and myopathy risk [30, 31]. However, previous reports on variants such as in *SLCO1B1* may be influenced by publication bias, with positive findings more likely to be reported than null results [32]. By using a genome‐wide PGS approach in a large cohort, our study mitigates this bias and provides a more comprehensive evaluation of genetic contribution [33]. Overall, these results suggest that while certain variants may contribute to short‐term statin‐associated muscle symptoms, the progressive decline in muscle function and mass among continuous statin users is largely independent of genetic susceptibility and more likely driven by lifestyle and/or compensatory physiological mechanisms.

Supporting healthy aging through lifestyle factors such as diet and physical activity may mitigate the metabolic disturbances through which statins contribute to muscle decline [34, 35]. In particular, the interaction between statin use and physical activity warrants careful consideration. Athletes and highly active individuals appear particularly sensitive to statin‐associated muscle symptoms, with prior reports indicating that athletes may report more complaints [36]. This sensitivity may help explain why we observed a decline in muscle mass among physically active statin users. Nevertheless, current evidence suggests that moderate exercise is generally safe under statin therapy, with no exacerbation of exercise‐induced muscle injury, and that the benefits of physical activity outweigh potential harms [36]. For instance, a study that compared statin users and non‐users found that both groups experienced similar increases in biomarkers of muscle damage, such as creatine kinase and lactate dehydrogenase, following moderate‐intensity exercise [37]. This suggests that statins do not amplify exercise‐induced injury. Overall, older statin patients should prioritize moderate‐intensity exercise to prevent muscle decline and benefit from additional cardiovascular protection [38].

Muscle decline is a key component of frailty, a multidimensional syndrome associated with falls, disability, hospitalization and mortality. The widely adopted Fried frailty criteria integrate five domains: unintentional weight loss, weakness (grip strength), poor endurance/exhaustion, slowness (gait speed) and low activity. Meeting three or more defines frailty, while one or two indicate prefrailty [39, 40]. Our study specifically examined one of these criteria, grip strength, focusing on trajectories of muscle function over time rather than applying fixed cut‐offs. Investigating the relationship between statin use and frailty risk could be the focus of future research.

Our study has several strengths. This includes a large sample size, adjustment for important confounders, longitudinal data with a median follow‐up period of 10 years, integration of genetic information and validation of BIA‐derived ALM against DXA‐derived ALM. However, some limitations should be acknowledged. First, statin use was self‐reported and confirmed during a verbal interview without information on dosage and duration of use before the recruitment. This may introduce misclassification, which would tend to bias results toward the null, potentially underestimating the true association between statin use and muscle outcomes. Second, our assessment of muscle health was limited to grip strength and ALM. Although both are widely accepted markers, they do not capture all dimensions of muscle quality, endurance, or power and the UK Biobank lacked objectively measured gait speed, an important component of frailty. Future studies incorporating more detailed functional evaluations could reinforce and extend these findings. Third, longitudinal analyses may be influenced by healthy survivor bias, as participants attending the repeat visits were generally healthier and more often male, which could attenuate long‐term associations. Finally, participants were of European ancestry, which may limit the generalizability of our findings to more diverse populations.

Our study indicates that continuous statin use is associated with a decline in muscle function and mass, irrespective of statin pharmacogenomic score. Muscle function and mass are closely linked to mobility, physical performance and health outcomes in aging populations. Preserving muscle health with age is essential for maintaining independence in daily activities and reducing the risk of frailty and falls. As statins are typically prescribed to individuals over 40 [19], the long‐term preservation of muscle function and mass is essential for maintaining independence in daily activities, preventing frailty and reducing the risk of falls in older adults. Future research should confirm the potential benefits of diet and physical activity for muscle function and mass among statin users. Specifically, examining the impact of different dietary patterns and exercise types on muscle health in individuals undergoing long‐term statin therapy will be crucial for promoting overall well‐being in aging populations and supporting the prevention of cardiovascular diseases.

## Ethics Statement

The UK Biobank project was approved by the ethics committee of the North West Multi‐center Research Ethics Committee. Additionally, the Regional Ethics Committee of Uppsala (Sweden) approved the study for data use. All participants provided written consent with the right to withdraw at any time.

## Conflicts of Interest

The authors declare no conflicts of interest.

## Supporting information


**Figure S1:** Flow chart.
**Table S1:** GWAS catalog SNP information for pharmacogenomic score calculation.
**Table S2:** The median of the food intake, used as a cut‐off for the diet quality score.
**Figure S2:** Standardized mean differences of covariates before and after one‐to‐one matching (unadjusted vs. adjusted) in the non‐imputed sample.
**Figure S3:** Standardized mean differences of covariates before and after one‐to‐one matching (unadjusted vs. adjusted) in the imputed sample.
**Table S3:** Sensitivity analyses of the cross‐sectional association of statin use with grip strength and appendicular lean mass.
**Table S4:** Association between continuous statin use and changes in grip strength and appendicular lean mass over 10‐year follow‐up after multiple imputations of missing values.
**Table S5:** Sensitivity analyses of grip strength and appendicular lean mass adjusting for glycaemic status and blood pressure medications.
**Table S6:** Sensitivity analyses of DXA‐derived appendicular lean mass and statin use.
**Figure S4:** Contribution of the selected SNPs to the pharmacogenomic score (PGS).
**Table S7:** Interaction of the pharmacogenomic score of statin response on the cross‐sectional association of statin use with grip strength and appendicular lean mass.
**Table S8:** Leave‐one‐SNP‐out sensitivity analysis to identify the most influential SNP in the pharmacogenomic score interaction in the cross‐sectional association between statin use and muscle‐related outcomes.
**Table S9:** Association between statin use and changes in grip strength and appendicular lean mass stratified by the pharmacogenomic score after multiple imputation of missing values.
**Figure S5:** Changes in grip strength and appendicular lean mass over time by statin use and diet quality.
**Figure S6:** Changes in grip strength and appendicular lean mass over time by statin use and physical activity.
**Table S10:** Characteristics of the study sample of the longitudinal analysis.

## Data Availability

This project was conducted using the UK Biobank resource under application number 30172. All the data that support the findings of this study are available from the UK Biobank. Permissions are required to gain access to the UK Biobank data resources, subject to a successful registration and application process. Further information can be found on the UK Biobank website (www.ukbiobank.ac.uk).
