# Supplementary material for: Statin Use Is Associated With a Decline in Muscle Function and Mass Over Time, Irrespective of Statin Pharmacogenomic Score
Source: J Cachexia Sarcopenia Muscle. 2025 Nov 20;16(6):e70132. doi: 10.1002/jcsm.70132 (PMC12634476; doi:10.1002/jcsm.70132)
Supplement: Supplementary file 1 — Figure S1: Flow chart. Table S1: GWAS catalog SNP information for pharmacogenomic score calculation. Table S2: The median of the food intake, used as a cut‐off for the diet quality score. Figure S2: Standardized mean differences of covariates before and after one‐to‐one matching (unadjusted vs. adjusted) in the non‐imputed sample. Figure S3: Standardized mean differences of covariates before and after one‐to‐one matching (unadjusted vs. adjusted) in the imputed sample. Table S3: Sensitivity analyses of the cross‐sectional association of statin use with grip strength and appendicular lean mass. Table S4: Association between continuous statin use and changes in grip strength and appendicular lean mass over 10‐year follow‐up after multiple imputations of missing values. Table S5: Sensitivity analyses of grip strength and appendicular lean mass adjusting for glycaemic status and blood pressure medications. Table S6: Sensitivity analyses of DXA‐derived appendicular lean mass and statin use. Figure S4: Contribution of the selected SNPs to the pharmacogenomic score (PGS). Table S7: Interaction of the pharmacogenomic score of statin response on the cross‐sectional association of statin use with grip strength and appendicular lean mass. Table S8: Leave‐one‐SNP‐out sensitivity analysis to identify the most influential SNP in the pharmacogenomic score interaction in the cross‐sectional association between statin use and muscle‐related outcomes. Table S9: Association between statin use and changes in grip strength and appendicular lean mass stratified by the pharmacogenomic score after multiple imputation of missing values. Figure S5: Changes in grip strength and appendicular lean mass over time by statin use and diet quality. Figure S6: Changes in grip strength and appendicular lean mass over time by statin use and physical activity. Table S10: Characteristics of the study sample of the longitudinal analysis. [file JCSM-16-e70132-s001.pdf]

Supplementary Material

Statin use is associated with a decline in muscle function and mass over time, irrespective of statin pharmacogenomic score

Mélissa Gentreau[1, *], Mahitab Sakr[1], Salahuddin Mohammed[1], Ahmed M Alsehli[1,2], Olga E Titova[3], Gull Rukh[1], Helgi B Schiöth[1]

[1] Functional Pharmacology and Neuroscience, Department of Surgical Sciences, Uppsala University, Uppsala, Sweden

[2] King Abdulaziz University, Department of Physiology, Faculty of Medicine, Al Ehtifalat St., 21589, Jeddah, Kingdom of Saudi Arabia

[3] Medical Epidemiology, Department of Surgical Sciences, Uppsala University, Uppsala, Sweden

Table of contents

Supplementary Figure 1. Flow chart.. 3

Supplementary Table 1. GWAS catalog SNP information for pharmacogenomic score calculation......... 4

Supplementary Table 2. The median of the food intake, used as a cut-off for the diet quality score. 9

Supplementary Figure 2. Standardized mean differences of covariates before and after one-to-one matching (unadjusted vs adjusted) in the non-imputed sample. 10

Supplementary Figure 3. Standardized mean differences of covariates before and after one-to-one matching (unadjusted vs adjusted) in the imputed sample. 11

Supplementary Table 3. Sensitivity analyses of the cross-sectional association of statin use with grip strength and appendicular lean mass. 12

Supplementary Table 4. Association between continuous statin use and changes in grip strength and appendicular lean mass over 10-year follow-up after multiple imputations of missing values. 13

Supplementary Table 5. Sensitivity analyses of grip strength and appendicular lean mass adjusting for glycemic status and blood pressure medications. 14

Supplementary Table 6. Sensitivity analyses of DXA-derived appendicular lean mass and statin use... 15

Supplementary Figure 4. Contribution of the selected SNPs to the pharmacogenomic score (PGS). 16

Supplementary Table 7. Interaction of the pharmacogenomic score of statin response on the cross-sectional association of statin use with grip strength and appendicular lean mass. 17

Supplementary Table 8. Leave-one-SNP-out sensitivity analysis to identify the most influential SNP in the pharmacogenomic score interaction in the cross-sectional association between statin use and muscle-related outcomes. 18

Supplementary Table 9. Association between statin use and changes in grip strength and appendicular lean mass stratified by the pharmacogenomic score after multiple imputation of missing values. 20

Supplementary Figure 5. Changes in grip strength and appendicular lean mass over time by statin use and diet quality... 21

Supplementary Figure 6. Changes in grip strength and appendicular lean mass over time by statin use and physical activity. 22

Supplementary Table 10. Characteristics of the study sample of the longitudinal analysis. 23

Supplementary References 24

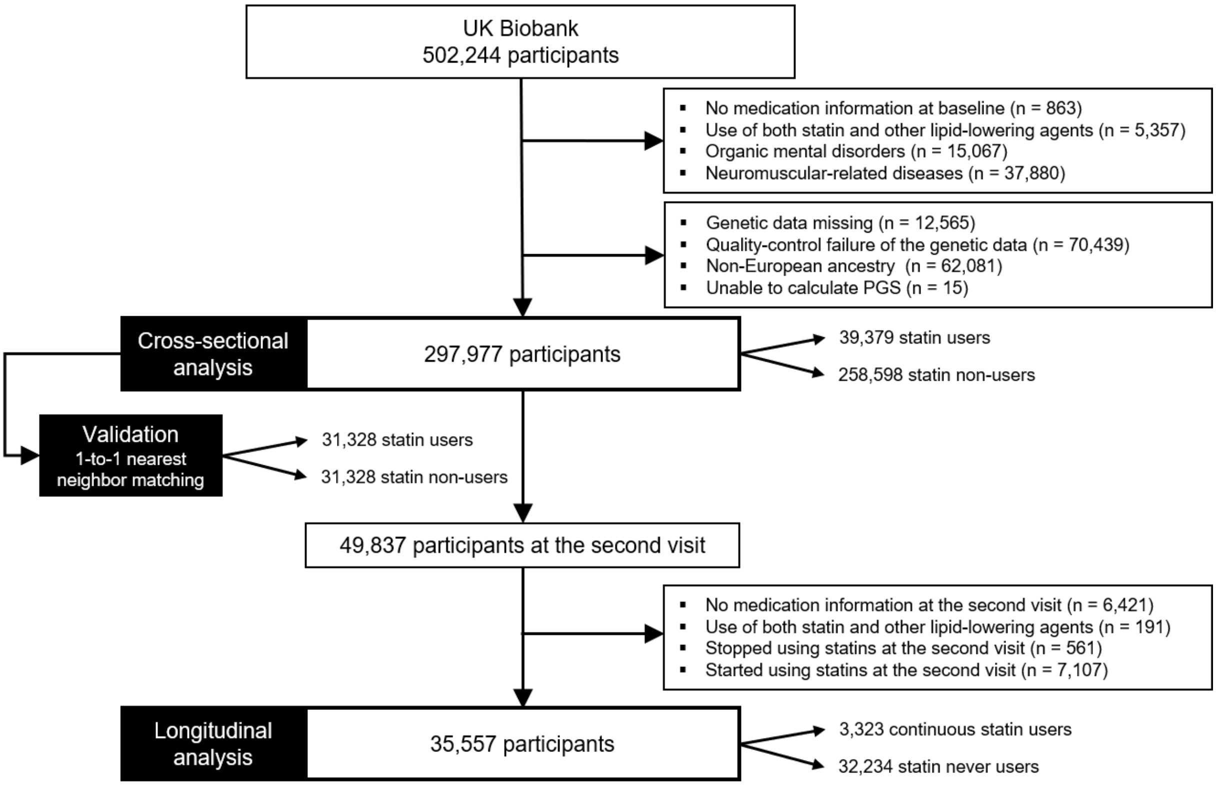

Supplementary Figure 1. Flow chart

Supplementary Table 1. GWAS catalog SNP information for pharmacogenomic score calculation.

PUBMED ID	DISEASE/TRAIT	SAMPLE SIZE	REGION	CHR ID	CHR POS	MAPPED GENE	SNPs	P-VALUE	PVALUE MLOG	OR/β	95% CI (TEXT)	PLATFORM
31220337	Atorvastatin-induced myopathy	28 European ancestry cases, 2,501 controls	8q21.2	8	83834281	LINC01419 - TPM3P3	rs11780883	1e-06	6	4.5454545	[2.27-9.09]	Illumina [654642] (imputed)
31220337	Atorvastatin-induced myopathy	28 European ancestry cases, 2,501 controls	19p12	19	22207269	ZNF676	rs34312380	3e-06	5.523	4.7619047	[2.17-11.11]	Illumina [654642] (imputed)
31220337	Atorvastatin-induced myopathy	28 European ancestry cases, 2,501 controls	5p15.31	5	7538189	ADCY2	rs4256319	3e-06	5.523	50	[8.33-∞]	Illumina [654642] (imputed)
35652242	AUC of simvastatin acid	229 European ancestry individuals	12p12.1	12	21178615	SLCO1B1	rs4149056	2e-17	16.699	0.518*	unit increase	Illumina [136688]
21386754	Cerivastatin-induced rhabdomyolysis	168 European ancestry cases, 636 European ancestry controls, 17 cases, 96 controls	1q43	1	237826822	RYR2	rs2819742	2e-07	6.698970004	2.08	[1.59-2.78]	Illumina [292461]
25214527	Creatine kinase levels in statin users	3,412 European ancestry individuals	19q13.32	19	45317925	CKM	rs11559024	4e-16	15.39794001	0.501	[0.38-0.62] unit decrease	Illumina [3232779] (imputed)
25214527	Creatine kinase levels in statin users	3,412 European ancestry individuals	19q13.42	19	54249685	LILRB5	rs2361797	2e-10	9.698970004	0.08	[0.055-0.105] unit increase	Illumina [3232779] (imputed)
26838040	Creatine kinase levels in statin users	5,207 Icelandic ancestry individuals	19q13.42	19	54255239	LILRB5	rs393600	9e-10	9.045757491	0.113	unit increase	Illumina [28300000] (imputed)
28043905	Drug-induced liver injury (statins)	59 European ancestry cases, 10,588 European ancestry controls	18q22.1	18	66962261	RNU6-1037P - MIR5011	rs116561224	7e-09	8.15490196	5.4	3.0-9.5	Illumina [505740] (imputed)
25089948	Response to simvastatin treatment (PCSK9 protein level change)	562 European ancestry individuals	3q13.2	3	113327793	CFAP44	rs13064411	8e-08	7.096910013	0.142	[NR] unit increase	Illumina [570422]
27587472	Response to statins (HDL cholesterol change)	16,769 European ancestry individuals	16q13	16	56955678	HERPUD1 - CETP	rs247616	9e-13	12.04575749	0.0071	[0.0090-0.0130] unit increase	Affymetrix, Illumina, Perlegen [up

												to 2500000] (imputed)
31969989	Response to statins (LDL cholesterol change)	28,616 European ancestry individuals, 1,205 African ancestry individuals, 2,350 East Asian ancestry individuals, 2,703 Hispanic/Latino individuals	19q13.32	19	44908822	APOE	rs7412	1e-78	78	0.068	[0.06-0.076] unit decrease	Affymetrix [13250765] (imputed)
25350695	Response to statins (LDL cholesterol change)	18,596 European ancestry individuals	6q25.3	6	160589086	LPA	rs10455872	7e-44	43.15490 196	0.052	[0.044-0.060] unit increase	Affymetrix, Illumina, Perlegen [at least 2500000] (imputed)
31969989	Response to statins (LDL cholesterol change)	28,616 European ancestry individuals, 1,205 African ancestry individuals, 2,350 East Asian ancestry individuals, 2,703 Hispanic/Latino individuals	1p13.3	1	109274570	CELSR2	rs7528419	1e-17	17	0.019	[0.015-0.023] unit decrease	Affymetrix [13250765] (imputed)
22331829	Response to statins (LDL cholesterol change)	6,989 European ancestry individuals	4q22.1	4	88117930	ABCG2	rs1481012	2e-15	14.69897	5.1	[3.34-6.86] % decrease	Illumina [814418]
22331829	Response to statins (LDL cholesterol change)	6,989 European ancestry individuals	1p32.3	1	55039974	PCSK9	rs11591147	5e-09	8.301029 996	5	[3.43-6.57] mg/dL decrease	Illumina [814418]
31969989	Response to statins (LDL cholesterol change)	28,616 European ancestry individuals, 1,205 African ancestry individuals, 2,350 East Asian ancestry individuals, 2,703 Hispanic/Latino individuals	19p13.2	19	11097306	LDLR	rs67337506	3e-08	7.522878 745	0.012	[0.0081-0.0159] unit decrease	Affymetrix [13250765] (imputed)

31969989	Response to statins (LDL cholesterol change)	28,616 European ancestry individuals, 1,205 African ancestry individuals, 2,350 East Asian ancestry individuals, 2,703 Hispanic/Latino individuals	2p24.1	2	21048451	APOB - TDRD15	rs1713222	5e-08	7.301029996	0.013	[0.0091-0.0169] unit decrease	Affymetrix [13250765] (imputed)
25350695	Response to statins (LDL cholesterol change)	18,596 European ancestry individuals	4p13	4	41488438	LIMCH1	rs7696430	8e-08	7.096910013	0.014	[0.0081-0.0199] unit increase	Affymetrix, Illumina, Perlegen [at least 2500000] (imputed)
22331829	Response to statins (LDL cholesterol change)	6,989 European ancestry individuals	6p22.3	6	16161194	MYLIP - MRPL42P2	rs6924995	5e-07	6.301029996	4.1	[2.73-5.47] mg/dL increase	Illumina [814418]
22331829	Response to statins (LDL cholesterol change)	6,989 European ancestry individuals	9q22.1	9	88925144	MIR4289 - PCNPP2	rs1875620	7e-07	6.15490196	2.8	[1.62-3.98] mg/dL increase	Illumina [814418]
25350695	Response to statins (LDL cholesterol change)	18,596 European ancestry individuals	4q31.3	4	153730876	RNF175	rs981844	1e-06	6	0.009	[0.0051-0.0129] unit increase	Affymetrix, Illumina, Perlegen [at least 2500000] (imputed)
31969989	Response to statins (LDL cholesterol percent change)	28,616 European ancestry individuals, 1,205 African ancestry individuals, 2,350 East Asian ancestry individuals, 2,703 Hispanic/Latino individuals	12p12.1	12	21204777	SLCO1B1	rs58310495	7e-12	11.15490196	0.069	[0.049-0.089] unit increase	Affymetrix [13250765] (imputed)
31220337	Simvastatin-induced myopathy	85 European ancestry cases, 2,501 controls	10q26.3	10	132440647	LINC03068	rs61865606	3e-07	6.522878745	2.564103	[1.39-4.76]	Illumina [654642] (imputed)
31220337	Simvastatin-induced myopathy	85 European ancestry cases, 2,501 controls	4p16.2	4	5634585	EVC2	rs140854723	1e-06	6	66.1	[10.6-414.0]	Illumina [654642] (imputed)

31220337	Simvastatin-induced myopathy	85 European ancestry cases, 2,501 controls	17p13.2	17	4495991	SPNS3 - SPNS2-AS1	rs333114	2e-06	5.698970004	3.22	[1.72-6.05]	Illumina [654642] (imputed)
31220337	Simvastatin-induced myopathy	85 European ancestry cases, 2,501 controls	7p12.3	7	48686597	ABCA13 - LINC02838	rs7779564	2e-06	5.698970004	3.37	[1.82-6.22]	Illumina [654642] (imputed)
31220337	Simvastatin-induced myopathy	85 European ancestry cases, 2,501 controls	17q21.32	17	47735928	TBX21	rs72648866	4e-06	5.397940009	3.37	[2.13-5.34]	Illumina [654642] (imputed)
31220337	Simvastatin-induced myopathy	85 European ancestry cases, 2,501 controls	10p13	10	12308135	CDC123 - RN7SL198P	rs10795948	5e-06	5.301029996	2.1276596	[1.54-2.94]	Illumina [654642] (imputed)
31220337	Simvastatin-induced myopathy	85 European ancestry cases, 2,501 controls	10q24.1	10	96947432	LCOR	rs184787123	6e-06	5.22184875	15.8	[4.64-53.9]	Illumina [654642] (imputed)
31220337	Simvastatin-induced myopathy	85 European ancestry cases, 2,501 controls	15q24.2	15	75740948	DNM1P35 - PPIAP47	rs148352615	7e-06	5.15490196	3.94	[2.23-6.97]	Illumina [654642] (imputed)
31220337	Simvastatin-induced myopathy	85 European ancestry cases, 2,501 controls	8p23.1	8	11028343	XKR6	rs117119573	8e-06	5.096910013	9.07	[1.96-41.93]	Illumina [654642] (imputed)
31220337	Simvastatin-induced myopathy	85 European ancestry cases, 2,501 controls	6p24.1	6	11754825	ADTRP	rs116168042	9e-06	5.045757491	2.76	[1.71-4.46]	Illumina [654642] (imputed)
31220337	Simvastatin-induced myopathy	85 European ancestry cases, 2,501 controls	6q15	6	88769294	RNGTT	rs6454721	9e-06	5.045757491	4	[1.85-8.33]	Illumina [654642] (imputed)
35543701	Statin intolerance or statin-associated muscle symptoms	819 European ancestry cases, 8,365 European ancestry controls, 49 Asian ancestry cases, 546 Asian ancestry controls, 14 Black cases, 282 Black controls, 12 cases, 530 controls	1q32.1	1	201148730	TMEM9	rs6667912	4e-08	7.397940009	1.33	[1.2-1.48]	Illumina [8921030] (imputed)
35543701	Statin intolerance or statin-associated muscle symptoms	819 European ancestry cases, 8,365 European ancestry controls, 49 Asian ancestry cases, 546 Asian ancestry	20q13.32	20	58072670	LINC01742 - CIMIP1	rs76443348	3e-07	6.522878745	2.55	[1.78-3.66]	Illumina [8921030] (imputed)

		controls, 14 Black cases, 282 Black controls, 12 cases, 530 controls										
35543701	Statin intolerance or statin-associated muscle symptoms	819 European ancestry cases, 8,365 European ancestry controls, 49 Asian ancestry cases, 546 Asian ancestry controls, 14 Black cases, 282 Black controls, 12 cases, 530 controls	2q24.1	2	154457782	GALNT13-AS1 - RNA5SP107	rs17815112	4e-07	6.397940009	0.77	[0.7-0.85]	Illumina [8921030] (imputed)
35543701	Statin intolerance or statin-associated muscle symptoms	819 European ancestry cases, 8,365 European ancestry controls, 49 Asian ancestry cases, 546 Asian ancestry controls, 14 Black cases, 282 Black controls, 12 cases, 530 controls	2q22.3	2	147667570	RNU6-715P - RNA5SP106	rs7564037	6e-07	6.22184875	0.57	[0.46-0.71]	Illumina [8921030] (imputed)
31220337	Statin-induced myopathy	128 European ancestry cases, 2,501 controls	5q23.3	5	128209813	SLC12A2 - FBN2	rs55902659	5e-06	5.301029996	2.2727273	[1.52-3.23]	Illumina [654642] (imputed)
31220337	Statin-induced myopathy	128 European ancestry cases, 2,501 controls	14q22.3	14	55255145	COX5AP1 - FBXO34-AS1	rs79860430	8e-06	5.096910013	2.59	[1.75-3.83]	Illumina [654642] (imputed)
31220337	Statin-induced myopathy (severe)	32 European ancestry cases, 2,501 controls	3p21.31	3	45114350	CDCP1	rs73089338	2e-07	6.698970004	4.63	[2.7-7.96]	Illumina [654642] (imputed)
31220337	Statin-induced myopathy (severe)	32 European ancestry cases, 2,501 controls	13q21.31	13	64923076	LINC00355 - LGMNP1	rs28447350	5e-07	6.301029996	3.66	[2.23-6.0]	Illumina [654642] (imputed)
31220337	Statin-induced myopathy (severe)	32 European ancestry cases, 2,501 controls	8p23.3	8	666484	ERICH1	rs2247256	2e-06	5.698970004	6.25	[0.023-16.667]	Illumina [654642] (imputed)

* The β was not reported in the GWAS catalog and was thus calculated from Table 1 of the original article.

Supplementary Table 2. The median of the food intake, used as a cut-off for the diet quality score.

Food intake	Coding	Median (IQR)	
		Women	Men
Cooked vegetable intake	heaped tablespoon/day	2 (1)	2 (1)
Salad / raw vegetable intake		2 (2)	1 (2)
Fresh fruit intake	pieces/day	2 (2)	2 (2)
Dried fruit intake		0 (1)	0 (1)
Oily fish intake	0: Never<br>1: Less than once a week<br>2: Once a week<br>3: 2-4 times a week<br>4: 5-6 times a week<br>5: Once or more daily	2 (1)	2 (1)
Processed meat intake		1 (1)	2 (2)
Beef intake		1 (1)	1 (1)
Lamb/mutton intake		1 (0)	1 (1)
Pork intake		1 (0)	1 (1)
Cheese intake		3 (1)	3 (1)

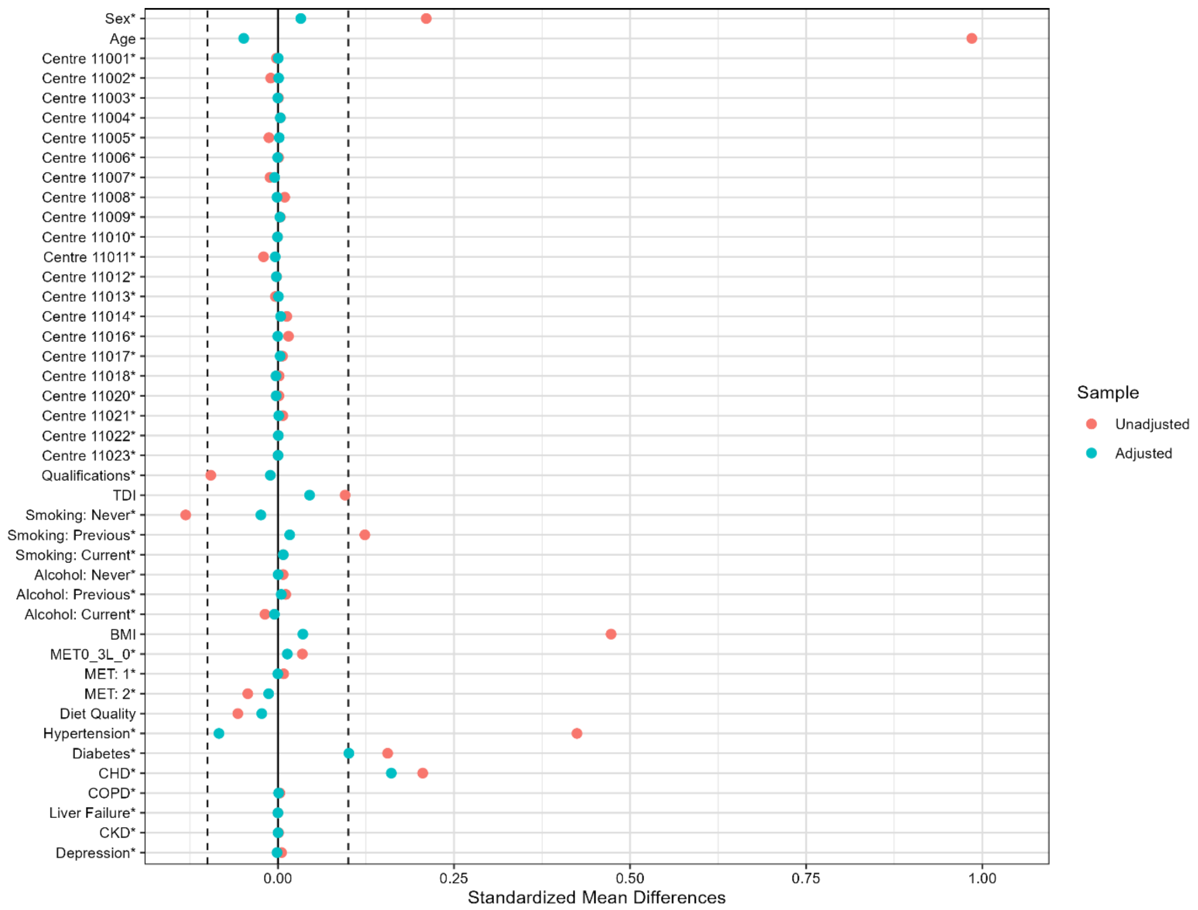

Supplementary Figure 2. Standardized mean differences of covariates before and after one-to-one matching (unadjusted vs adjusted) in the non-imputed sample.

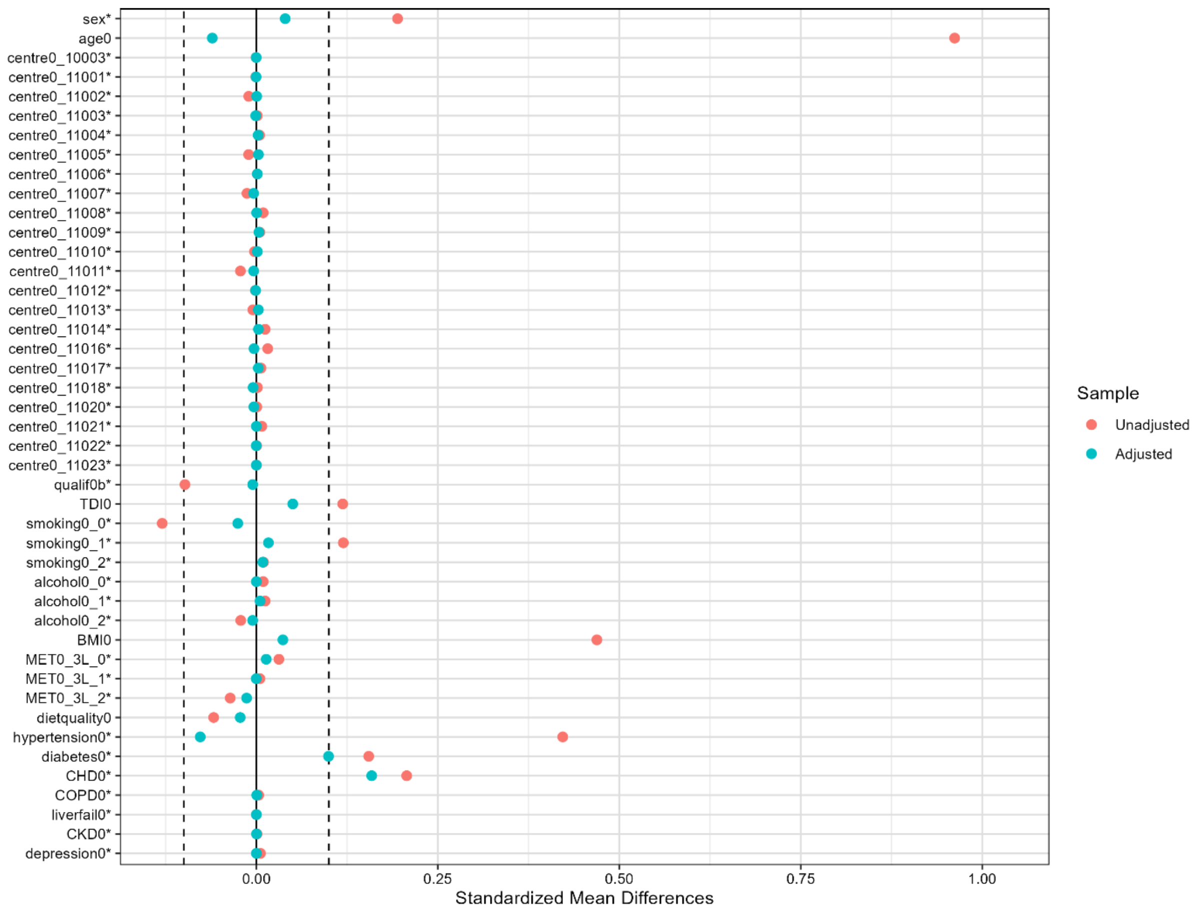

Supplementary Figure 3. Standardized mean differences of covariates before and after one-to-one matching (unadjusted vs adjusted) in the imputed sample.

Supplementary Table 3. Sensitivity analyses of the cross-sectional association of statin use with grip strength and appendicular lean mass.

Outcome	n	β	95% Confidence Interval Lower	95% Confidence Interval Upper	P value
**Grip Strength (kg)**					
Imputed sample	296,527	-0.701	-0.874	-0.527	3e-15
Propensity score without imputation	56,150	-0.579	-0.828	-0.331	5e-06
Propensity score with imputation	78,296	-0.518	-0.726	-0.309	1e-06
**Appendicular lean mass (kg)**					
Imputed sample	293,459	-0.195	-0.222	-0.167	<1e-16
Propensity score without imputation	56,150	-0.169	-0.21	-0.127	2e-15
Propensity score with imputation	76,750	-0.184	-0.219	-0.148	<1e-16

Linear model adjusted for assessment center, age, sex, education, TDI, smoking and drinking status, BMI, MET score, diet quality score, history of depression, hypertension, diabetes, coronary heart disease, liver failure/cirrhosis, chronic obstructive airway diseases/COPD, renal/kidney failure.

Supplementary Table 4. Association between continuous statin use and changes in grip strength and appendicular lean mass over 10-year follow-up after multiple imputations of missing values.

Outcome		95% Confidence Interval		
	β	Lower	Upper	P value
**Grip Strength (kg)**				
Statin use	3.686	2.013	5.359	2e-05
Time	-1.247	-1.304	-1.19	<1e-16
Statin use * Time	-0.356	-0.51	-0.202	6e-06
**Appendicular lean mass (kg)**				
Statin use	0.356	0.141	0.57	0.001
Time	-0.077	-0.085	-0.069	<1e-16
Statin use * Time	-0.040	-0.062	-0.018	3e-04

Statin use is defined as continuous use between baseline and follow-up versus never (reference). Linear model adjusted for assessment center, age, sex, education, TDI, smoking and drinking status, BMI, MET score, diet quality score, history of depression, hypertension, diabetes, coronary heart disease, liver failure/cirrhosis, chronic obstructive airway diseases/COPD, renal/kidney failure.

Supplementary Table 5. Sensitivity analyses of grip strength and appendicular lean mass adjusting for glycemic status and blood pressure medications.

Outcome	β	95% Confidence Interval Lower	Upper	P value
		**Cross-sectional analysis**		
Grip Strength (kg)				
Statin use	-0.578	-0.789	-0.368	7e-08
Appendicular lean mass (kg)				
Statin use	-0.183	-0.216	-0.149	<1e-16
		**Longitudinal analysis**		
Grip Strength (kg)				
Statin use	3.36	1.462	5.258	5e-04
Time	-1.27	-1.336	-1.205	<1e-16
Statin use * Time	-0.328	-0.503	-0.154	2e-04
Appendicular lean mass (kg)				
Statin use	0.536	0.294	0.778	1e-05
Time	-0.078	-0.087	-0.069	<1e-16
Statin use * Time	-0.056	-0.081	-0.031	8e-06

Linear model adjusted for assessment center, age, sex, education, TDI, smoking and drinking status, BMI, MET score, diet quality score, history of depression, coronary heart disease, liver failure/cirrhosis, chronic obstructive airway diseases/COPD, renal/kidney failure, hypertension or blood pressure medication, glycemic status (normoglycemia, prediabetes, diabetes).

Supplementary Table 6. Sensitivity analyses of DXA-derived appendicular lean mass and statin use.

Outcome		95% Confidence Interval		
	β	Lower	Upper	P value
**Cross-sectional analysis**				
Appendicular lean mass (kg)				
Statin use	-0.234	-0.326	-0.143	5e-07
**Longitudinal analysis**				
Appendicular lean mass (g)				
Statin use	0.447	0.112	0.783	0.009
Time	-0.128	-0.168	-0.088	4e-10
Statin use * Time	-0.163	-0.277	-0.048	0.005

Linear model adjusted for assessment center, age, sex, education, TDI, smoking and drinking status, BMI, MET score, diet quality score, history of depression, hypertension, diabetes, coronary heart disease, liver failure/cirrhosis, chronic obstructive airway diseases/COPD, renal/kidney failure.

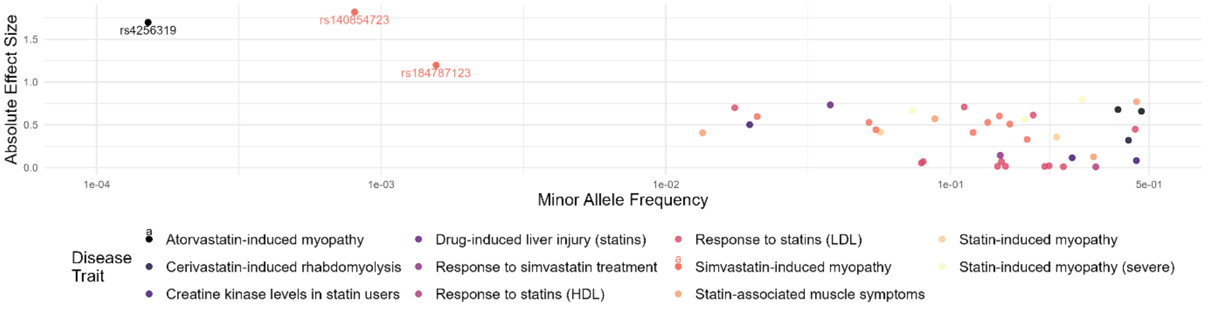

Supplementary Figure 4. Contribution of the selected SNPs to the pharmacogenomic score (PGS).

SNPs are represented according to their MAF in function of their absolute effect size. They are color-coded based on their associated disease traits, highlighting key contributors to genetic susceptibility of statin response.

Supplementary Table 7. Interaction of the pharmacogenomic score of statin response on the cross-sectional association of statin use with grip strength and appendicular lean mass.

Outcome		95% Confidence Interval		
	β	Lower	Upper	P value
**Grip Strength (kg)**				
Statin use	-0.119	-0.743	0.506	0.71
PGS	2.565	-0.846	5.976	0.141
Statin use * PGS	-9.36	-19.021	0.301	0.058
**Appendicular lean mass (kg)**				
Statin use	-0.1	-0.199	-0.001	0.047
PGS	0.776	0.236	1.315	0.005
Statin use * PGS	-1.428	-2.961	0.105	0.068

Linear model adjusted for SNP chip, the 10 genetic principal components, assessment center, age, sex, education, TDI, smoking and drinking status, BMI, MET score, diet quality score, history of depression, hypertension, diabetes, coronary heart disease, liver failure/cirrhosis, chronic obstructive airway diseases/COPD, renal/kidney failure.

Supplementary Table 8. Leave-one-SNP-out sensitivity analysis to identify the most influential SNP in the pharmacogenomic score interaction in the cross-sectional association between statin use and muscle-related outcomes.

Outcome	β	95% Confidence Interval		P value
		Lower	Upper	
**Grip Strength (kg)**				
Statin use * (PGS - rs10455872)	-8.143	-17.516	1.231	0.089
Statin use * (PGS - rs10795948)	-9.184	-18.631	0.263	0.057
Statin use * (PGS - rs11559024)	-8.267	-17.879	1.346	0.092
Statin use * (PGS - rs11591147)	-8.259	-17.656	1.138	0.085
Statin use * (PGS - rs116168042)	-8.082	-17.501	1.337	0.093
Statin use * (PGS - rs116561224)	-8.715	-18.185	0.755	0.071
Statin use * (PGS - rs117119573)	-8.057	-17.429	1.316	0.092
Statin use * (PGS - rs11780883)	-7.932	-17.816	1.951	0.116
Statin use * (PGS - rs13064411)	-8.224	-17.609	1.161	0.086
Statin use * (PGS - rs140854723)	-8.057	-17.436	1.321	0.092
Statin use * (PGS - rs1481012)	-7.398	-17.004	2.208	0.131
Statin use * (PGS - rs148352615)	-8.209	-17.618	1.199	0.087
Statin use * (PGS - rs1713222)	-8.071	-17.443	1.301	0.091
Statin use * (PGS - **rs17815112**)	**-6.294**	-16.391	3.803	**0.222**
Statin use * (PGS - rs184787123)	-8.238	-17.622	1.145	0.085
Statin use * (PGS - rs1875620)	-8.401	-18.012	1.211	0.087
Statin use * (PGS - rs2247256)	-8.912	-18.969	1.145	0.082
Statin use * (PGS - rs2361797)	-8.267	-17.879	1.346	0.092
Statin use * (PGS - rs247616)	-8.063	-17.435	1.309	0.092
Statin use * (PGS - rs2819742)	-7.737	-17.234	1.760	0.110
Statin use * (PGS - rs333114)	-8.340	-17.879	1.198	0.087
Statin use * (PGS - rs34312380)	-7.482	-17.406	2.441	0.139
Statin use * (PGS - rs393600)	-7.727	-17.108	1.654	0.106
Statin use * (PGS - rs4256319)	-8.090	-17.463	1.284	0.091
Statin use * (PGS - rs55902659)	-8.537	-18.011	0.937	0.077
Statin use * (PGS - rs58310495)	-7.839	-17.212	1.535	0.101
Statin use * (PGS - rs61865606)	-7.827	-17.251	1.596	0.104
Statin use * (PGS - rs6454721)	-7.164	-16.759	2.430	0.143
Statin use * (PGS - rs6667912)	-8.153	-17.545	1.238	0.089
Statin use * (PGS - rs67337506)	-8.090	-17.462	1.282	0.091
Statin use * (PGS - rs6924995)	-7.215	-16.870	2.439	0.143
Statin use * (PGS - rs72648866)	-8.158	-17.591	1.276	0.090
Statin use * (PGS - rs73089338)	-7.652	-17.165	1.861	0.115
Statin use * (PGS - rs7412	-8.162	-17.536	1.212	0.088
Statin use * (PGS - rs7528419	-8.165	-17.537	1.207	0.088
Statin use * (PGS - rs7564037	-9.173	-18.674	0.329	0.058
Statin use * (PGS - rs76443348	-8.078	-17.459	1.302	0.091
Statin use * (PGS - rs7696430	-8.082	-17.455	1.290	0.091
Statin use * (PGS - rs7779564	-7.354	-16.876	2.168	0.130

Statin use * (PGS - rs79860430	-8.550	-17.958	0.857	0.075
Statin use * (PGS - rs981844)	-8.058	-17.431	1.314	0.092
Statin use * (PGS - rs79128089)	-8.267	-17.879	1.346	0.092
**Appendicular lean mass (kg)**				
Statin use * (PGS - rs10455872)	-1.239	-2.727	0.248	0.102
Statin use * (PGS - rs10795948)	-1.331	-2.831	0.168	0.082
Statin use * (PGS - rs11559024)	-1.288	-2.814	0.237	0.098
Statin use * (PGS - rs11591147)	-1.220	-2.711	0.271	0.109
Statin use * (PGS - rs116168042)	-1.224	-2.718	0.271	0.109
Statin use * (PGS - rs116561224)	-1.259	-2.762	0.243	0.101
Statin use * (PGS - rs117119573)	-1.256	-2.743	0.231	0.098
Statin use * (PGS - rs11780883)	-1.745	-3.313	-0.176	0.029
Statin use * (PGS - rs13064411)	-1.269	-2.759	0.220	0.095
Statin use * (PGS - rs140854723)	-1.285	-2.774	0.203	0.091
Statin use * (PGS - rs1481012)	-1.520	-3.045	0.004	0.051
Statin use * (PGS - rs148352615)	-1.289	-2.781	0.204	0.091
Statin use * (PGS - rs1713222)	-1.260	-2.747	0.227	0.097
Statin use * (PGS - **rs17815112**)	**-0.301**	-1.904	1.302	**0.713**
Statin use * (PGS - rs184787123)	-1.216	-2.705	0.273	0.109
Statin use * (PGS - rs1875620)	-1.419	-2.945	0.106	0.068
Statin use * (PGS - rs2247256)	-1.030	-2.627	0.566	0.206
Statin use * (PGS - rs2361797)	-1.288	-2.814	0.237	0.098
Statin use * (PGS - rs247616)	-1.258	-2.746	0.229	0.097
Statin use * (PGS - rs2819742)	-1.250	-2.757	0.257	0.104
Statin use * (PGS - rs333114)	-1.331	-2.845	0.182	0.085
Statin use * (PGS - rs34312380)	-1.111	-2.686	0.463	0.167
Statin use * (PGS - rs393600)	-1.195	-2.684	0.293	0.116
Statin use * (PGS - rs4256319)	-1.237	-2.724	0.250	0.103
Statin use * (PGS - rs55902659)	-1.376	-2.880	0.127	0.073
Statin use * (PGS - rs58310495)	-1.226	-2.714	0.261	0.106
Statin use * (PGS - rs61865606)	-1.257	-2.753	0.238	0.099
Statin use * (PGS - rs6454721)	-1.381	-2.903	0.141	0.075
Statin use * (PGS - rs6667912)	-1.369	-2.859	0.122	0.072
Statin use * (PGS - rs67337506)	-1.263	-2.750	0.224	0.096
Statin use * (PGS - rs6924995)	-1.127	-2.659	0.405	0.149
Statin use * (PGS - rs72648866)	-1.269	-2.765	0.228	0.097
Statin use * (PGS - rs73089338)	-1.268	-2.777	0.242	0.100
Statin use * (PGS - rs7412	-1.243	-2.730	0.244	0.101
Statin use * (PGS - rs7528419	-1.264	-2.751	0.224	0.096
Statin use * (PGS - rs7564037	-1.168	-2.676	0.340	0.129
Statin use * (PGS - rs76443348	-1.261	-2.749	0.228	0.097
Statin use * (PGS - rs7696430	-1.258	-2.745	0.229	0.097
Statin use * (PGS - rs7779564	-1.118	-2.629	0.393	0.147
Statin use * (PGS - rs79860430	-1.273	-2.766	0.219	0.095
Statin use * (PGS - rs981844)	-1.258	-2.745	0.229	0.097
Statin use * (PGS - rs79128089)	-1.288	-2.814	0.237	0.098

Supplementary Table 9. Association between statin use and changes in grip strength and appendicular lean mass stratified by the pharmacogenomic score after multiple imputation of missing values.

Outcome		95% Confidence Interval		
	β	Lower	Upper	P value
**Changes in Grip Strength (kg)**				
Low PGS				
Statin use	1.889	-1.296	5.074	0.245
Time	-1.23	-1.34	-1.12	<1e-16
Statin use * Time	-0.223	-0.516	0.07	0.136
Medium PGS				
Statin use	3.987	0.618	7.356	0.02
Time	-1.277	-1.389	-1.165	<1e-16
Statin use * Time	-0.37	-0.68	-0.059	0.02
High PGS				
Statin use	3.695	0.589	6.802	0.02
Time	-1.265	-1.374	-1.157	<1e-16
Statin use * Time	-0.347	-0.632	-0.062	0.017
**Changes in Appendicular lean mass (kg)**				
Low PGS				
Statin use	0.706	0.298	1.113	7e-04
Time	-0.074	-0.089	-0.059	<1e-16
Statin use * Time	-0.076	-0.118	-0.034	4e-04
Medium PGS				
Statin use	0.545	0.114	0.975	0.013
Time	-0.08	-0.095	-0.065	<1e-16
Statin use * Time	-0.057	-0.101	-0.013	0.011
High PGS				
Statin use	0.39	-0.007	0.786	0.054
Time	-0.079	-0.094	-0.064	<1e-16
Statin use * Time	-0.044	-0.084	-0.004	0.032

Statin use is defined as continuous use between baseline and follow-up versus never (reference). Linear model adjusted for SNP chip, 10 genetic principal components, assessment center, age, sex, education, Townsend deprivation index, smoking and drinking status, BMI, MET score, diet quality score, history of depression, hypertension, diabetes, coronary heart disease, liver failure/cirrhosis, chronic obstructive airway diseases/COPD, renal/kidney failure.

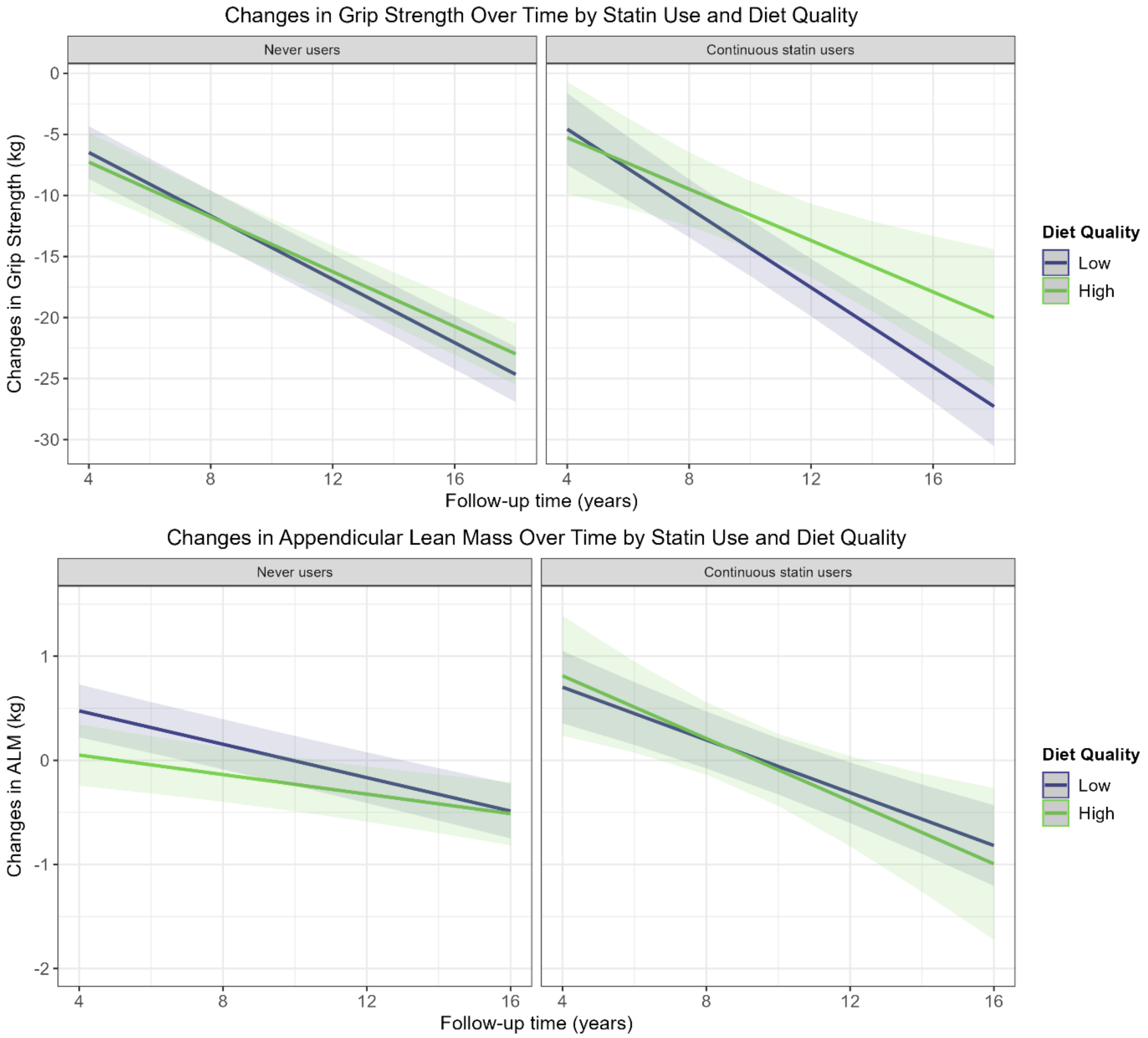

Supplementary Figure 5. Changes in grip strength and appendicular lean mass over time by statin use and diet quality. Predicted changes in grip strength (top) and appendicular lean mass (bottom) with a 95% confidence interval were obtained from the linear model adjusted for assessment center, age, sex, education, TDI, smoking and drinking status, BMI, MET score, history of depression, hypertension, diabetes, coronary heart disease, liver failure/cirrhosis, chronic obstructive airway diseases/COPD, renal/kidney failure. Low diet quality was defined as being in the lowest tertile of the diet quality score at both baseline and follow-up. High diet quality was defined as being in the highest tertile of diet quality score at both baseline and follow-up.

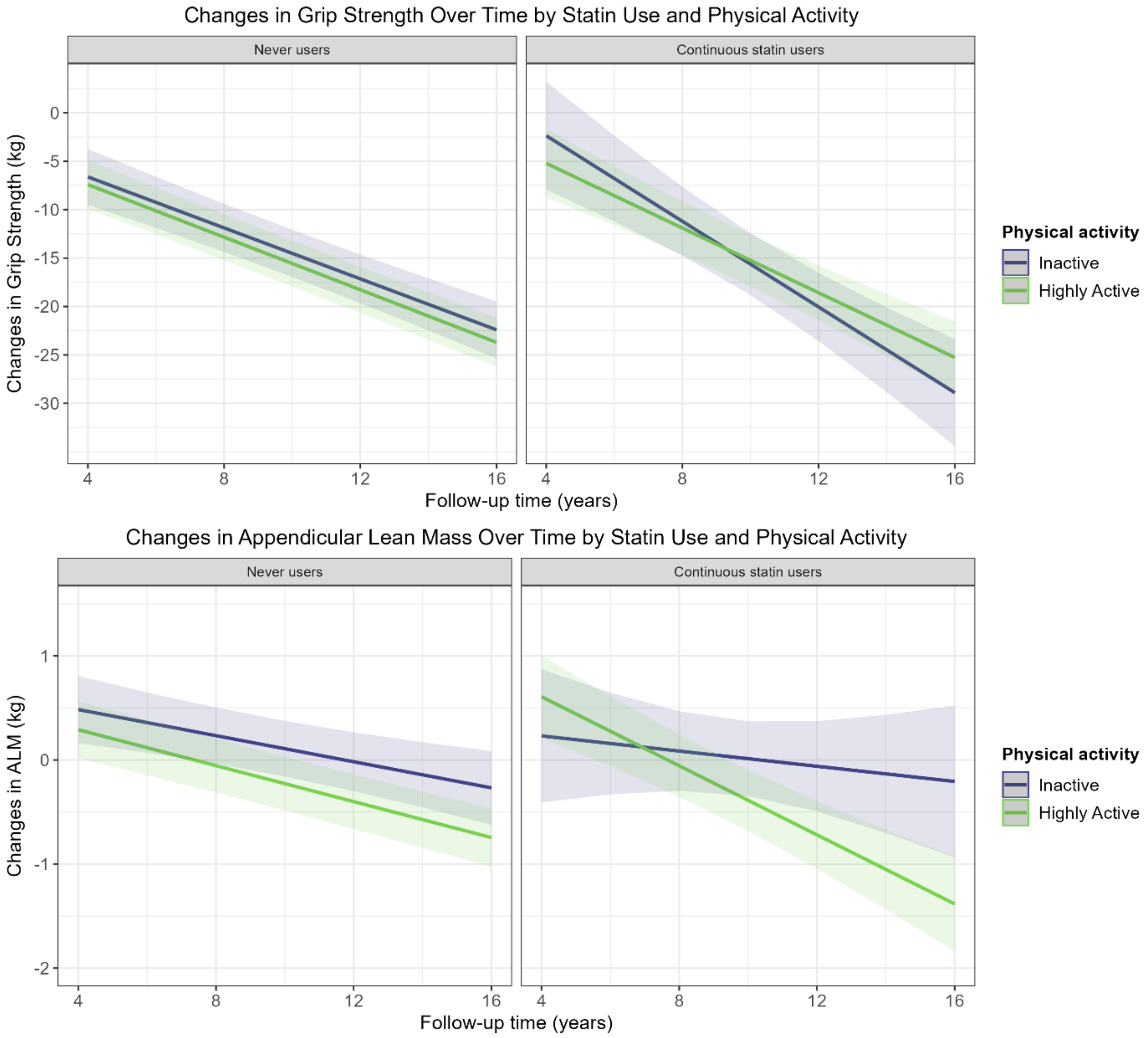

Supplementary Figure 6. Changes in grip strength and appendicular lean mass over time by statin use and physical activity. Predicted changes in grip strength (top) and appendicular lean mass (bottom) with a 95% confidence interval were obtained from the linear model adjusted for assessment center, age, sex, education, TDI, smoking and drinking status, BMI, diet quality score, history of depression, hypertension, diabetes, coronary heart disease, liver failure/cirrhosis, chronic obstructive airway diseases/COPD, renal/kidney failure. Inactive was defined as being in the low physical activity level at both baseline and follow-up. Highly active was defined as being in the high physical activity level at both baseline and follow-up.

Supplementary Table 10. Characteristics of the study sample of the longitudinal analysis.

Characteristics<br>Mean (SD) or n (%)	All Sample<br>n = 35,557	Statin non-users<br>n = 32,234	Statin users<br>n = 3,323
Age	54.1 (7.5)	53.5 (7.4)	60 (5.9)
Sex (Men)	16541 (46.5)	14139 (43.9)	2402 (72.3)
College or university degree	16293 (45.8)	15024 (46.6)	1269 (38.2)
TDI*	-2.7 (3.2)	-2.7 (3.2)	-2.7 (3.2)
BMI (kg/m²)	26.3 (4.2)	26.1 (4.1)	28.6 (4.3)
MET Score			
<600 min/week	5314 (14.9)	4730 (14.7)	584 (17.6)
600 to <1200 min/week	5511 (15.5)	4947 (15.3)	564 (17)
≥ 1200 min/week	19228 (54.1)	17544 (54.4)	1684 (50.7)
Smoking status			
Never	22184 (62.4)	20526 (63.7)	1658 (49.9)
Previous	11250 (31.6)	9809 (30.4)	1441 (43.4)
Current	2064 (5.8)	1846 (5.7)	218 (6.6)
Alcohol status			
Never	712 (2)	645 (2)	67 (2)
Previous	703 (2)	624 (1.9)	79 (2.4)
Current	34135 (96)	30959 (96)	3176 (95.6)
Diet quality	5.5 (1.7)	5.5 (1.7)	5.4 (1.7)
Hypertension	5641 (15.9)	3750 (11.6)	1891 (56.9)
Diabetes	546 (1.5)	119 (0.4)	427 (12.8)
Coronary heart diseases	696 (2)	101 (0.3)	595 (17.9)
Chronic obstructive airway diseases/COPD	32 (0.1)	27 (0.1)	5 (0.2)
Liver failure/cirrhosis	9 (0)	8 (0)	1 (0)
Renal/Kidney failure	6 (0)	2 (0)	4 (0.1)
Depression	1580 (4.4)	1405 (4.4)	175 (5.3)
Medications for blood pressure	3946 (11.1)	2035 (6.3)	1911 (57.5)
Medications for diabetes	122 (0.3)	25 (0.1)	97 (2.9)
HbA1c			
< 42 mmol/mol	32642 (91.8)	30029 (93.2)	2613 (78.6)
42-47 mmol/mol	671 (1.9)	401 (1.2)	270 (8.1)
≥ 48 mmol/mol	398 (1.1)	121 (0.4)	277 (8.3)

* Median (interquartile range)

Supplementary References

S1. Guan Z-W, Wu K-R, Li R, Yin Y, Li X-L, Zhang S-F *et al.* Pharmacogenetics of statins treatment: Efficacy and safety. *Journal of Clinical Pharmacy and Therapeutics* 2019;**44**:858–867.

S2. Sudlow C, Gallacher J, Allen N, Beral V, Burton P, Danesh J *et al.* UK biobank: an open access resource for identifying the causes of a wide range of complex diseases of middle and old age. *PLoS Med* 2015;**12**:e1001779.

S3. Gentreau M, Rukh G, Miguet M, Clemensson LE, Alsehli AM, Titova OE *et al.* The Effects of Statins on Cognitive Performance Are Mediated by Low-Density Lipoprotein, C-Reactive Protein, and Blood Glucose Concentrations. *J Gerontol A Biol Sci Med Sci* 2023;**78**:1964–1972.

S4. Cruz-Jentoft AJ, Bahat G, Bauer J, Boirie Y, Bruyère O, Cederholm T *et al.* Sarcopenia: revised European consensus on definition and diagnosis. *Age Ageing* 2019;**48**:16–31.

S5. Kirwan R, Mazidi M, Butler T, Perez de Heredia F, Lip GYH, Davies IG. The association of appendicular lean mass and grip strength with low-density lipoprotein, very low-density lipoprotein, and high-density lipoprotein particle diameter: a Mendelian randomization study of the UK Biobank cohort. *Eur Heart J Open* 2024;**4**:oeae019.

S6. Choi SW, Mak TS-H, O'Reilly PF. Tutorial: a guide to performing polygenic risk score analyses. *Nat Protoc* 2020;**15**:2759–2772.

S7. Benjamin EJ, Blaha MJ, Chiuve SE, Cushman M, Das SR, Deo R *et al.* Heart Disease and Stroke Statistics-2017 Update: A Report From the American Heart Association. *Circulation* 2017;**135**:e146–e603.

S8. Austin PC. Balance diagnostics for comparing the distribution of baseline covariates between treatment groups in propensity-score matched samples. *Stat Med* 2009;**28**:3083–3107.
